# Pasteur and Jenner

**Published:** 1893-01-21

**Authors:** William Tebb

**Affiliations:** Devonshire Club, St. James's, London


### EDITOR'S LETTER-BOX.

[Our correspondents are reminded that prolixity is a great bar to publication, and that brevity of style and conciseness of statement greatly facilitate early insertion.]

### PASTEUR AND JENNER.

SIR,—The celebration of the seventieth birthday of Pasteur in Paris has been the occasion for extravagant laudation of a man whose title to fame rests upon medical discoveries, which, while extending human knowledge, have done nothing to mitigate suffering or prevent disease. Before a distinguished audience, at the Palace of the Sorbonne, M. Dupuy, the Minister of Public Instruction, pronounced a eulogy on Pasteur as a man "who was able to concentrate his thoughts on one subject." . . . "Ferments and virus," he said, "are living beings; vaccine is an attenuated virus, the basis of medicine is the artificial attenuation of virus, and thus the microbe treatment is founded." Sir Joseph Lister, who represented English bacteriologists, in allusion to Pasteur's studies of rabies, addressed the discoverer, and said that "with the exception of certain ignorant people, everybody now recognises the greatness of that which you have accomplished against this terrible malady. . . You can, therefore understand that medicine and surgery are eager on this great occasion to offer you the profound homage of their admiration and of their gratitude." Would it be believed that the particular discoveries upon which Pasteur's reputation rests are hotly disputed in every country in Europe, and their utility denied by the most eminent pathologists? The carefully tabulated statistics of Professor Michel Peter, of Paris, show that the alleged preventive of rabies is purely mythical and powerless to prevent the disease, while the inoculated virus is not seldom attended with deadly results. But history repeats itself. In looking over old volumes of Hansard, I find a report of the debate on the award proposed to be given to Jenner in 1802. The Right Hon. H. Addington, Chancellor of the Exchequer (who afterwards became Prime Minister) said: "Whatever sum of money the House might vote as a future reward of Jenner's merit, he had already received the highest reward in the approbation, the unanimous approbation of the House of Commons, an approbation most richly deserved since it was the result of the greatest, or one of the most important, discoveries to human society that was made since the creation of man. That the value of the discovery was without example and beyond all calculation were points not to be contested, for they were made out by convincing evidence." Nearly a century's experience has shown vaccination to be no preventive of small-pox, but a source of incalculable mischief in the spread of the most terrible diseases, including syphilis and leprosy. But such is the credulity of mankind when weighted with medical or ecclesiastical authority that it may now take another half century to get rid of the Pasteurian incubus.

WILLIAM TEBB.

Devonshire Club, St. James's, London,

January 7th, 1893.

[This letter is referred to elsewhere —ED. *T.H.*]

